# Measurement of the effectiveness of *Clonostachys rosea* in reducing *Fusarium* biomass on wheat straw

**DOI:** 10.1007/s13353-024-00906-8

**Published:** 2024-09-14

**Authors:** Tomasz Kulik, Kinga Treder, Marta Rochoń, Dariusz Załuski, Paweł Sulima, Jacek Olszewski, Katarzyna Bilska, Georgina Elena, Tadeusz Kowalski

**Affiliations:** 1 Department of Botany and Evolutionary Ecology, University of Warmia and Mazury in Olsztyn, Plac Lodzki 1, 10-718 Olsztyn, Poland; 2 Department of Agroecosystems, Faculty of Environmental Management and Agriculture, University of Warmia and Mazury in Olsztyn, Plac Łódzki 3, 10-718 Olsztyn, Poland; 3 Department of Genetics, Plant Breeding and Bioresource Engineering, Plac Lodzki 3, 10-724 Olsztyn, Poland; 4 Experimental Education Unit, University of Warmia and Mazury in Olsztyn, Pl. Łódzki 1, 10-727 Olsztyn, Poland; 5 Wageningen Plant Research, Wageningen University and Research, P.O. Box 16, 6700 AA Wageningen, the Netherlands; 6 Department of Forest Ecosystems Protection, University of Agriculture in Krakow, Al. 29 Listopada 46, 31-425 Krakow, Poland

**Keywords:** *Clonostachys rosea*, *Fusarium*, Wheat straw, qPCR assays

## Abstract

The survival and growth of plant pathogens on crop residues are key factors facilitating the dynamics of crop diseases. Spores (e.g., perithecia, and chlamydospores) and mycelium of pathogenic fungi overwinter on harvest residues, such as straw, and serve as initial inoculum infecting crops in the next growing season. Therefore, targeting overwintering fungi is essential to attaining effective disease control. Beneficial microorganisms offer advantages in controlling pathogens through their ability to colonize and exploit different environmental niches. In this study, we applied qPCR assays to explore the biocontrol performance of locally isolated strains of *Clonostachys* against various *Fusarium* pathogens. We proved that prior colonization of wheat straw by *Fusarium* spp. can be effectively reduced by *Clonostachys rosea*. We demonstrated that the efficiency of *C. rosea* to reduce *Fusarium* inoculum appears to remain at a similar level for most studied strains regardless of the target pathogen and the level of colonization of substrates by pathogens. Efficient performance of local *C. rosea* strains identifies possible targets for future strategies to control Fusarium diseases in cereals. Our study also highlights the challenge in sequence-based determination of *C. rosea,* which is crucial for the efficient selection of beneficial strains for biocontrol purposes.

**Supplementary Information:**

The online version contains supplementary material available at 10.1007/s13353-024-00906-8.

## Introduction

Plant diseases are a major global concern since they have a negative impact on the production of food crops, which affects the social and political stability of societies (Ristaino et al. 2021). Nowadays, plant diseases cause 10–15% annual loss of major crops worldwide, with around 70–80% of those diseases being caused by phytopathogenic fungi (Peng et al. 2021). In addition, outbreak risks are increased by globalization and climate change, which encourage the spread of pathogens in new areas (Singh et al. 2023). Nowadays, an increase in crop production along with a reduction in food loss due to pathogens are among the main current challenges to the global food supply (Ristaino et al. 2021). Cereal crops play a key role in agriculture because of their essential role in the human diet and their steadily increasing importance in the feed industry. However, even with modern agricultural practices, fungal pathogens continue to blight cereal production worldwide, with estimated yield losses of 15–20% (Różewicz et al. 2021).

To control fungal diseases, a combination of various cultural practices with the selection of appropriate cultivars is recommended (Pirgozliev et al. 2003; Sinha et al. 2022; Figueroa et al. 2018). More efficient suppression of the disease is often achieved through the application of fungicides (Steinberg & Gurr 2020). However, there are significant environmental issues related to their widespread use in agriculture. Firstly, fungicides can affect many biological systems and decrease microbial diversity in fields (Zubrod et al. 2019; Ma et al. 2021). Secondly, their extensive use in cereal production leads to the emergence of fungicide resistance, which becomes a serious threat to agriculture and medicine (Fisher et al. 2022). In addition, some fungicides have been linked to direct negative health outcomes. For example, azoles, which are often used to control cereal pathogens can target various CYP (cytochrome P 450) enzymes in humans and can inhibit enzymes of the steroidogenic pathway and disrupt the synthesis of steroid hormones (Draskau & Svingen 2022).

Fungi of the genus *Fusarium* are amongst the most common pathogens of cereals causing significant yield losses in many parts of the cereal-growing regions worldwide. They infect plants at the seedling stage leading to the development of Fusarium Crown Root (FCR) disease, characterized by leaf sheath or stem base browning and whiteheads (Chakraborty et al. 2006). Another disease, fusarium head blight (FHB), or ear rot, occurs during flowering and has emerged as the main threat to the global production of grains (Khan et al. 2020). Besides yield loss, the disease results in contamination of the grains with mycotoxins posing a severe health risk for both humans and animals (Munkvold 2017). Both FCR and FHB can be caused by a range of *Fusarium* species, which distribution may vary seasonally and annually (Ji et al. 2019; Karlsson et al. 2021). Fusarium composition can also differ between regions and cereal hosts, although numerous epidemiological surveys showed that *Fusarium graminearum* is currently the most often isolated cause of FHB in Asia, Europe, and North America (Del Ponte et al. 2022). From an epidemiological perspective, spores (e.g., perithecia, and chlamydospores) and mycelium overwintering on crop residues, such as straw, are the main reservoir of plant pathogenic *Fusarium* spp. (Drakopoulos et al. 2021). Spores, which are produced by these structures serve as inoculum infecting crops in the next growing season (Moonjely et al. 2023). Therefore, it appears that reducing the amounts of overwintering fungi is essential to attaining effective disease control.

A large body of research has demonstrated the contribution of beneficial microorganisms, such as well-studied *Trichoderma* fungi*,* to the protection of cereal crops against *Fusarium* pathogens under both in vitro and field conditions (Luongo et al. 2005; Modrzewska et al. 2022). Other studies highlighted the promising efficacy of *Clonostachys rosea* in controlling fungal diseases. The fungus exhibits strong mycoparasitic activity against numerous fungal plant pathogens, nematodes, and insects (Sun et al. 2020); however, only a limited number of studies demonstrated beneficial traits of *C. rosea* against *Fusarium* pathogens of cereals (Luongo et al. 2005; Hue et al. 2009; Köhl et al. 2015; Khairullina et al. 2023) and did not evaluate the performance of *C. rosea* in reducing pathogen inoculum on natural substrates. We hypothesized that *C. rosea* exhibits efficient performance against a set of *Fusarium* pathogenic to wheat. The biocontrol efficiency of the selected strains was assessed based on their ability to reduce pathogen inoculum on wheat straw occupied by *Fusarium* spp. We asked the following questions: (i) How effectively can *C. rosea* reduce pathogen inoculum in infected straw? (ii) Can we consider local strains of *C. rosea* in future strategies aiming at the development of biological agents against plant pathogens? We quantified the reduction of pathogen colonization in a total of 84 bioassays *Clonostachys* vs. *Fusarium* in an attempt to solve these interesting questions.

## Materials and methods

### Fungal strains and culture condition

**Fig. 1:**
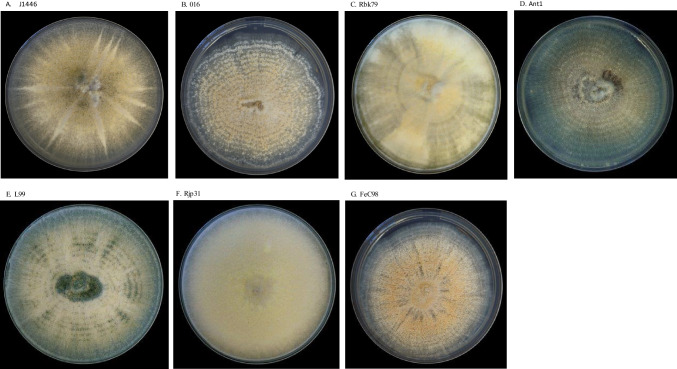
*Clonostachys* strains grown on PDA medium

A total of 7 mycoparasitic strains were used in this study. Local strains (*n* = 5) were obtained from two academic fungal collections: Collection of the Department of Forest Ecosystems Protection, University of Agriculture in Kraków and Collection of the Department of Botany and Evolutionary Ecology, University of Warmia and Mazury in Olsztyn (Table 1). They were isolated from different hosts/substrates in various geographic locations in Poland and were initially assigned as *Clonostachys*-like based on culture morphology on PDA (Fig. 1A–G). Commercially available strain J1446 was included for comparative analyses. This strain was recovered from the biofungicide Prestop (Verdera Oy, Finland) by plating a powder sample (0.1 g) on a PDA plate. We also included *C. rosea* 016 strain due to its documented high activity against *F. graminearum* (Gimeno et al. 2021).

**Table 1:** List of *Clonostachys* strains used to study their mycoparasitic activity

Fungal strain*, species	Host	Geographical origin, isolation date	Comments/References
J1446, *C. rosea*	Soil	FI, 1997	Active ingredient of the biofungicide Prestop (Verdera Oy, Finland)
016^1^, *C. rosea*	unknown	NL, 2004	Strain effective against *F. graminearum, F. avenaceum and F. verticillioides* (Palazzini et al. 2013)
Rjp31^2^, *C. rosea*	*Fraxinus pennsylvanica*	Kraków, PL, 2016	Local strain
Rbk79^2^, *C. rosea*	*Fagus sylvatica*	Kraków, PL, 2019	Local strain
L99^3^, *C. rosea*	Compost bin	Lipina, PL, 2023	Local strain
Ant1^3^, *C. rosea*	anthill	Olsztyn, PL, 2023	Local strain
FeC98^2^, *C. solani*	*Fraxinus excelsior*	Limanowa, PL, 2018	Local strain

^*^Fungal collections: ^1^Culture collection of the BU Biointeractions & Plant Health of the Plant Sciences Group of Wageningen University and Research, ^2^Collection of the Department of Forest Ecosystems Protection, University of Agriculture in Kraków, ^3^Collection of the Department of Botany and Evolutionary Ecology, University of Warmia and Mazury in Olsztyn (Olsztyn, Poland)

*FI* Finland, *NL* Netherlands, *PL* Poland

The biocontrol efficiency of these strains was studied against different strains of *Fusarium* (Table 2). Target *Fusarium* strains were chosen to represent major *Fusarium* species associated with FCR or FHB of small-grain cereals in Europe (Bottalico & Perrone 2002). For comparison, we also included *Fusarium oxysporum* isolated from wheat. *Fusarium oxysporum* is not involved in cereal diseases but has been recorded as an endophyte in cereals (Asim et al. 2022).

**Table 2:** List of *Fusarium* spp. used to study mycoparasitic efficiency *Clonostachys* strains

Fungal strain*, species	Host	Geographical origin, isolation date	Taxonomic confirmation
1001t^3^, CBS 138561^4^, *F. graminearum*	Wheat	PL, 2010	Phylogenomic approach, Kulik et al. 2022
CBS 128539^4^, *F. graminearum*	Wheat	BE, 2007	Phylogenomic approach, Kulik et al. 2022
S18-4^3^, *F. graminearum*	Soybean	PL, 2018	Phylogenomic approach, Kulik et al. 2022
0302^3^, CBS 139512^4^,* F. culmorum*	Wheat	PL, 2003	Species-specific qPCR, Bilska et al. 2018b
16/84/2^3^, *F. culmorum*	Wheat	PL, 2016	Species-specific qPCR, Bilska et al. 2018a
CBS 597.96^4^, *F. culmorum*	Wheat	unknown	Species-specific qPCR, Kulik 2011
16/89/2^3^, *F. avenaceum*	Wheat	PL, 2016	Species-specific qPCR, Bilska et al. 2018b
17/4/RO^3^,* F. avenaceum*	Wheat	PL, 2017	Species-specific qPCR, Bilska et al. 2018b
S22020KK^3^,* F. equiseti*	Soybean	PL, 2020	Żelechowski et al. 2021
06/301^3^, *F. langsethiae*	Wheat	PL, 2016	this study
CBS 412.86^4^, *F. sporotrichioides*	Juncus, stipe	DK, 1984	Kulik 2008
AR 2016–1^5^, *F. oxysporum*	Wheat	RU, 2016	this study

^*^Fungal collections: ^3^Collection of the Department of Botany and Evolutionary Ecology, University of Warmia and Mazury in Olsztyn (Olsztyn, Poland), ^4^CBS-KNAW—Westerdijk Fungal Biodiversity Institute (Utrecht, The Netherlands), ^5^ARRP—All-Russian Research Institute of Phytopathology, Bolshie Vyazyomy (Moscow, Russia)

*PL* Poland, *BE* Belgium, *NO* Norway, *DK* Denmark, *RU* Russia

### Extraction of DNA from *Clonostachys* strains and whole-genome sequencing

DNA from fungal strains was extracted with the use of the commercial DNA extraction kit (PureLink Genomic DNA Mini Kit, Invitrogen, Carlsbad, CA, USA) according to the manufacturer’s protocol. For DNA extraction, 0.1 g of mycelium scrapped with a sterile tip from fungi grown on PDA for 7 days at 28 °C. DNA from each isolate was quantified on a Qubit fluorometer using a Qubit dsDNA BR assay kit (Applied Biosystems, Foster City, CA, USA). Whole-genome sequencing was performed as previously described by Kulik et al. 2022. Briefly, genomes of all mycoparasitic strains were sequenced by Macrogen (Seoul, South Korea) on an Illumina HiSeq X Ten using a paired-end read length of 2 × 150 bp with an insert size of 350 bp. Libraries were prepared using TruSeq DNA PCR-free library preparation kit (Illumina, San Diego, CA, United States). All genomic data generated herein were deposited in the NCBI Sequence Read Archive under GenBank accession no. PRJNA1017172. The genomes were further assembled via SPAdes (v.3.13.2) (Nurk et al. 2013) with k-mer values of 21, 33, 55, 77, 99, and 127 and using the “careful” option to reduce mismatches.

### Taxonomic assignments of the strains

For taxonomic assignments, complete sequences of barcodes: *tef-1α* (translation elongation factor 1 alpha, XM_011321607), *top1* (topoisomerase I, XM_011328230), *rpb1* (XM_011318349), *rpb2* (RNA polymerase II genes, XM_011322661), *tub2* (beta-tubulin, XM_011329885), *pgk* (phosphoglycinecerate kinase, XM_011323364), *calm* (calmodulin, XM_011319438), *mcm7* (mini-chromosome maintenance proteins, XM_011328505), and ITS (nuclear ribosomal internal transcribed spacer, XR_893061) were first downloaded from the reference genome of *F. graminearum* PH-1 strain (Cuomo et al. 2007) with Geneious Prime software (Biomatters Ltd., New Zealand). Barcode genes were further retrieved from the genome assembly of studied strains based on a homology search for the corresponding sequences from the PH-1 strain with Geneious Prime software (Biomatters Ltd., New Zealand). For BLAST analyses, short sequences (500–800 bp) recommended for barcoding of fungi (Stielow et al. 2015; Lücking et al. 2020) were selected from each gene to maximize coverage of expected BLAST hits. Species determination was achieved through multiple sequence comparisons using the BLAST searches (Altschul et al. 1990) using thresholds of 99–100% nucleotide identity and ≥ 90% coverage of the query sequence length. The lack or limited number of entries from *Clonostachys* genus in GenBank database encouraged us to include more sequence data for comparative analyses. To achieve it, additional genomes of strains of closely related *C. rosea/C. solani* were subjected to analyses (Table 3). Whole genome sequencing, genome assembly, and extraction of barcode sequences for comparative analyses were retrieved from twelve additional genomes as described earlier. To determine intra/interspecies variability within and among *C. rosea* and *C. solani*, barcode sequences were compared through multiple sequence alignments. The analysis was performed with progressive Mauve (Darling et al. 2010) implemented in Geneious Prime software.

**Table 3:** List of strains used for taxonomic confirmation of *Clonostachys* strains through sequence comparisons

Fungal strain*, species	Host	Geographical origin, isolation date
BN074^1^, *C. rosea*	Apple tree	NL, 2019
BN148^1^, *C. rosea*	Apple tree	NL, 2019
BN149^1^, *C. rosea*	Apple tree	NL, 2019
247^1^, *C. rosea*	Onion	NL, 2004
017^1^, *C. rosea f. catenula*	unknown	NL, 2004
Rjs67^2^, *C. rosea*	*Fraxinus excelsior*	PL, Andrychów, 2018
Rsw58^2^, *C. solani*	*Picea abies*	PL, Białowieża, 2016
Rsw59^2^, *C. solani*	*Picea abies*	PL, Białowieża, 2016
Rsw68^2^, *C. solani*	*Picea abies*	PL, Białowieża, 2016
Rjs13^2^, *C. solani*	*Fraxinus excelsior*	PL, Miechów, 2017
Rjp26^2^, *C. solani*	*Fraxinus pennsylvanica*	PL, Kraków, 2016
Rps287^2^, *C. solani*	*Pinus sylvestris*	PL, Kraków, 2019

^*^Fungal collections: ^1^Culture collection of the BU Biointeractions & ^1^Plant Health of the Plant Sciences Group of Wageningen University and Research, ^2^Collection of the Department of Forest Ecosystems Protection, University of Agriculture in Kraków

*NL* Netherlands, *PL* Poland

### Bioassays

**Fig. 2:**
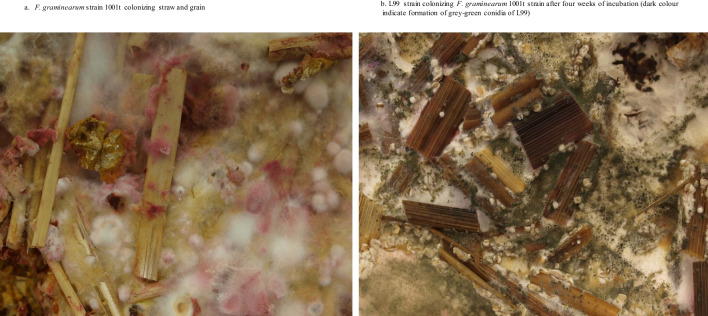
**a** *F. graminearum* strain 1001t colonizing straw and grain. **b** L99 strain colonizing *F. graminearum* 1001t strain after 4 weeks of incubation (dark color indicates formation of grey-green conidiaof L99)

To evaluate the performance of *C. rosea* in reducing pathogen inoculum on plant residues, we used wheat straw, which served as a substrate for the long-term growth of strains used in our experiments. An essential requirement to allow a high level of fungal colonization of the straw was the availability of a substantial amount of fungal biomass. To achieve it, we first pre-cultured *Clonostachys* and *Fusarium* strains in separate 15 cm diameter sterile Petri dishes with PDA medium (potato dextrose agar) (A&A Biotechnology, Gdynia, Poland) covered with 25 g of sterile rice grain. The use of a PDA medium facilitated the rapid production of fungal biomass. Covering agar surface with grains reduced water loss due to evaporation. After 7 days of incubation at 21 °C, sterilized 3- to 5-cm-long wheat straw pieces were placed on rice overgrown by individual strains of *Fusarium* to allow efficient colonization of straw. After 2 weeks of incubation at 21 °C, agar plugs (4–6 cm diameter) with grain and straw overgrown by pathogen hyphae (Fig. 2a, b) were transferred with a sterile scalpel to rice overgrown by single *Clonostachys* strains. A single sample involved a bioassay of two strains: *Clonostachys* strain vs. *Fusarium* strain. Control samples involved cultures with single *Fusarium* strains. Each bioassay was repeated twice with three triplicate measurements in each experiment. After 3 weeks of co-culture at 21 °C, agar plugs with grain and straw were transferred to 50 ml plastic tubes and frozen for DNA extraction.

### DNA extraction from fungi

Samples of inoculated wheat straw (0.1 g) were homogenized (30 s at speed 6.0 m/s) on a FastPrep-24 instrument (MP Bio-medicals, Solon, OH, USA) in tubes with 1-mm silica spheres (Lysing matrix C, MP Biomedicals) for DNA extraction. DNA was isolated with a commercial DNA extraction kit (PureLink Genomic DNA Mini Kit, Invitrogen, Carlsbad, CA, USA). To quantify *Fusarium* biomass in rice substrate, DNA from rice grain (0.1 g) overgrown by fungi was extracted with the same procedure as described for wheat straw.

### Quantification of fungi by qPCR

Two qPCR assays targeting single-copy genes in fungi were used to quantify *Fusarium* (Sohlberg et al. 2022) and *C. rosea* (Gimeno et al. 2019) in straw and grain (Table 4). Primers were synthesized by Sigma-Aldrich (Germany), while probes were ordered from ABI PRISM Primers and TaqMan Probe Synthesis Service. The qPCR reagents were used as follows: 2 µl of extracted DNA, 12 µl H2O, 6 µM of primer each, 1.7 µM of probe, and Real-Time 2 × PCR Master Mix Probe (Taq DNA polymerase 1 U µl -1, reaction buffer (2 ×), MgCl_2_ (10 mM), dNTP mix (0.5 mM each), stabilizers), 0.2 µl ROX 50 (A&A Biotechnology, Gdynia, Poland). The reaction setup was performed with the epMotion 5070 automated pipetting system (Eppendorf, Hamburg, Germany). All PCR amplifications were carried out in a QuantStudio™ 3 Real-Time PCR (Applied Biosystems, Foster City, CA, USA) with a final volume of 20 µl. The qPCR reaction condition was 95 °C for 20 s (95 °C for 3 s, 60 °C for 55 s) × 40. Each qPCR was prepared in at least three replicates. The amount of fungal DNA was calculated from Ct values using the standard curve. The amplification efficiency of the assay was determined based on fivefold dilutions of the DNA template extracted from pure fungal cultures of 1001t and Ant1 strains. The differences in *Fusarium* DNA quantities in bioassays were determined by comparing them to the control samples (*Fusarium-*only cultures) and assigned as follows: strong reduction (> 70%), moderate (40–70%), and weak reduction (up to 40%).

**Table 4:** The data of efficiency, linearity, and regression slope obtained from qPCR assays used in this study

qPCR assay	Primer/Probe and sequence (5′ to 3′)	Target gene	Specificity	Efficiency	Linearity (R2 value)	Regression slope	References
FusE	FusEF (forward) CTGGGTTCTTGACAAGCTCA FusER (reverse) CGGTGACATAGTAGCGAGGA FusEP (probe) TACCACGCTCACGCTCGGCT	*tef-1α*	*Fusarium* spp.	99.6%	0.977	− 3,333	Sohlberg et al. 2022
VTTact	VTTact-forward GGCCAGAGATTGTGTTGATGA VTTact-reverse ACAGGTTAGGCTCAATGCTC VTTact probe GAGGCTGGCAAGAGAGGTCAGTCAC	actin	*C. rosea*	103.5%	0.912	-3.240	Gimeno et al. 2019

### Statistical analyses

We evaluated mycoparasitic performance of *Clonostachys* strains based on their efficiency in reducing *Fusarium* DNA in bioassays. Calculated DNA amounts from controls (solely incubated *Fusarium* strains) and bioassays *Clonostachys* vs. *Fusarium* were performed based on the *t*-test for dependent samples (paired samples) at a significance level of *α* = 0.05. The *t*-test for dependent samples is a statistical test for comparing the means from two dependent populations or the difference between the means from two populations. Dependent samples occur when the subjects are paired up. Most often, this model is characterized by a selection of a random sample where each member is observed under two different conditions, before and after the experiment. The *t*-test is used when the differences are normally distributed. Normality was assessed using the Shapiro–Wilk test. Such analyses were performed using the STATISTICA 13.3 program (TIBCO, Palo Alto, CA, USA).

## Results

### Whole genome sequencing and taxonomic confirmation of the strains

In this study, we provided a total of 19 SRA (Sequence Read Archive) submissions, containing over 72 Gb of raw sequence data for resolving taxonomic uncertainties of studied strains. Multiple sequence analyses of *tef-1α, calm, top1, mcm7, rpb1, rpb2, tub2, pgk* genes, and ITS (Internal Transcribed Spacer) extracted from these genomic data were used to confirm the taxonomic status of the *Clonostachys*-like strains. Among them, all except one were identified as *C. rosea* based on BLAST searches of five barcodes: *tef-1α*, *tub2*, *rpb2*, *rpb1*, and ITS (Supplementary file 1). The remaining strain FeC98 was determined as *C. solani* using BLASTn analyses of *tub2* and *rpb2* genes (Supplementary file 1). Failure in resolving taxonomic issues using other barcodes resulted from a small fraction of sequences from *Clonostachys* spp. available in the GenBank database.

The official fungal barcode ITS region enabled the successful identification of *C. rosea*, but failed to confirm the identity of *C. solani,* probably due to inadequate or incorrect labeling of sequence entries in GenBank. Further multiple sequence comparisons of studied barcodes indicated satisfactory discriminating power of all these loci in differentiating strains of *C. rosea* and *C. solani* (Table 5). The strain FeC98 displayed the highest (99.7–100%) degree of nucleotide similarity to other *C. solani* strains. Similarly, the pattern of low levels of intraspecific variation was also evident in *C. rosea* for most barcode genes tested in this study (Table 5)*.* The taxonomic identity of the majority of *Fusarium* strains used in this study was confirmed in our previous studies using different species-specific qPCR assays or sequence-based approaches (Table 2). However, no published data is available with regard to two studied strains: 06/301 (*F. langsethiae)* and AR 2016–1 (*F. oxysporum).* To confirm their taxonomic status, we extracted nucleotide sequences *tef-1α* and* tub2* genes from previously sequenced genomes of these strains for BLASTn analyses. Assuming 100% identity match and ≥ 95% query coverage, BLAST searches against the NCBI database for strain 06/301 yielded hits to *F. langsethiae* using both *tef-1α* and *tub2* genes. For the strain AR 2016–1, BLASTn analyses of both *tef-1α* and *tub2* genes yielded hits to *F. oxysporum* (100% identity match and ≥ 95% query coverage) (Supplementary file 2).

**Table 5:** Intra- and interspecies identity between *C. rosea* and *C. solani* based on sequence comparisons

Barcode length (bp)	Intraspecies identity (%)		Max interspecies identity between two species (%)
	*C. rosea*^*1*^	*C. solani*^*2*^	
*tef-1α (580 bp)*	99.4–100	100	76.1
tub2 (897 bp)	97.6–100	99.7–100	96.7
top1 (670 bp)	99.1–100	99.7–100	96.1
rpb2 (765 bp)	99.6–100	100	95.9
rpb1 (815 bp)	99.6–100	99.8–100	95.6
pgk (813 bp)	99.8–100	99.9–100	97.8
mcm7 (674 bp)	99.4–100	100	96.1
calm (530 bp)	99.7–100	100	95.8
ITS (624 bp)	99.7–100	100	99.1

^1^Fungal strains: J1446, 016, Rjp31, Rbk79, L99, Ant1, 017, 247, BN074, BN148, BN149, Rjs67

^2^Fungal strains: FeC98, Rjs13, Rjp26, Rps287, Rsw58, Rsw59, Rsw68

### Quantification of *Fusarium* inoculum in straw and grain

We first assessed the colonization efficiency of straw and grain by strains of *Fusarium* using a qPCR (Sohlberg et al. 2022). The assays allowed the quantification of studied strains with an acceptable reaction efficiency (Table 4).

The level of colonization of straw differed among strains with a nearly ten-fold difference. Among studied strains, *F. culmorum* strain 0302 was quantified at the highest levels (17.4 ng; SD 12.7), while the lowest quantity (1.8 ng; SD 0.5) of the pathogen was determined in the case of *F. avenaceum* strain 16/8912 (Supplementary file 3). The colonization level of grain also showed considerable, nine-fold difference. *Fusarium oxysporum* strain Ar 2016–1 colonized grain at the highest levels (21.8 ng; SD 10.8), while the lowest load (2.4 ng; SD 0.5) of fungal inoculum was found for *F. sporotrichioides* strain CBS 412.86.

In addition, the type of substrate used in this study appeared to have varied impacts on colonization efficiency by *Fusarium* strains. Most straw samples colonized by *F. culmorum* and *F. graminearum* contained higher levels of fungal load than in grain. An opposite trend could be observed in the case of strains of *F. avenaceum* and *F. oxysporum,* which were quantified at considerably higher levels in grain than in straw.

### Quantification of *Fusarium* inoculum in bioassays 

We evaluated the biocontrol performance of *Clonostachys* strains based on their efficiency in reducing *Fusarium* inoculum. Among the total of 84 bioassays, a strong reduction (> 70%) of *Fusarium* quantity was found in most (*n* = 77) bioassays, as compared to positive controls (*Fusarium* strains solely incubated on the substrate) (Supplementary file 4). Among strains of *C. rosea,* three (J1446, Rjp-31, and 016) strongly decreased quantities of all tested *Fusarium* strains in straw. Moderate reduction was quantified in seven bioassays however, in three (L99 vs. 0302, L99 vs. 16/8912, and L99 vs. CBS 412.86) the reduction of pathogens was not significant. Correspondingly, a strong reduction of pathogen load was also found in most (*n* = 70) grain samples. Two strains (J1446 and Rbk79) showed a strong reduction of all twelve *Fusarium* strains. A moderate reduction of pathogen biomass was found in eight samples. A weak but not significant reduction of pathogen load was detected in four bioassays: L99 vs. S22020 KK, 016 vs. CBS 597.96, Rjp-31 vs. 1001t, and Rjp-31 vs. 0302. In two bioassays: Rjp-31 vs. CBS 597.96 and L99 vs. 1001t, a statistically significant increase of *Fusarium* quantity was found as compared to positive controls. Single *C. solani* strain FeC98 used in this study, showed high biocontrol performance against *Fusarium* fungi by its strong reduction of pathogen quantity in most tested bioassays.

To determine whether a less effective decrease of pathogen load might be linked to insufficient biocontrol efficiency*,* we quantified levels of *C. rosea* in bioassays showing low to large reduction of pathogens. Two local *C. rosea* strains were selected: L99 and Ant1. The strain L99 insufficiently decreased pathogen load in six samples, while Ant1 effectively reduced pathogens in all tested bioassays. The results of qPCR analyses showed that ineffective reduction of *Fusarium* was not associated with low colonization efficiency by *C. rosea*. The levels of L99 in three straw samples showing insufficient reduction of pathogens ranged from 28.5 to 46.5 ng (Table 6) and were even higher than in most (*n* = 16) straw samples with both L99 and Ant1 strains, where strong to moderate reduction of *Fusarium* spp. were found. Similarly, analysis of grain substrate did not indicate whether insufficient decrease of pathogens could be explained by the drastic reduction of L99 quantity, which ranged from 13.2 to 26.3 ng (Table 6) and was generally comparable to levels found in other bioassays with L99 (Supplementary file 3).

**Table 6:** Quantity of L99 and Ant1 strain in substrates colonized by *Fusarium* strains after three weeks of co-culture

*Fusarium* strain, species	Quantity of *C. rosea* (ng of DNA/SD) in straw samples		Quantity of *C. rosea* (ng of DNA/SD) in grain samples	
	L99	Ant1	L99	Ant1
1001t, *F. graminearum*	12,5/3,2	8,6/4,8	26,3/14,1°	9,0/3,2
CBS 128539, *F. graminearum*	20,0/9,6	16,2/3,5	11,6/4,5	13,5/4,7
S 18/4, *F. graminearum*	26,2/9,7	19,5/12,4	13,3/3,2	5,7/1,8
0302, *F. culmorum*	28,5/11,4°	17,1/17,3	13,6/7,7	21,1/5,0
16/84/2, *F. culmorum*	25,3/3,6	24,8/19,5	20,1/7,1	31,9/20,9
CBS 597.96, *F. culmorum*	29,8/16,1°	24,1/12,4	21,6/14,0	10,1/3,7
17/4 RO, *F. avenaceum*	13,6/2,6	6,9/3,9	29,0/7,0	20,3/12,2
16/8912, *F. avenaceum*	46,5/37,4°	22,6/18,3	21,7/15,4°	16,2/11,1
S22020 KK, *F. equiseti*	47,3/15,4	6,4/4,0	13,2/9,8°	23,1/14,6
06/301, *F. langsethiae*	14,3/4,5	26,0/9,3	11,0/2,1	3,1/1,2
CBS 412.86, *F. sporotrichioides*	33,1/14,4	20,5/14,0	12,4/3,2	10,1/4,1
Ar 2016–1, *F. oxysporum*	26,3/7,3	17,6/3,5	17,2/5,9	30,3/2,9

°Samples with less effective reduction of pathogen load

## Discussion

The dynamics of plant diseases are determined by the overwintering survival of fungi, which facilitates their invasive success between seasons. Plant residues left on the soil surface serve as substrate for pathogens, which spores are further spread to nearby plants, by wind and rain splash dispersal mechanisms (Drakopoulos et al. 2021). *Fusarium* pathogens employ this strategy to infect crops the next season (Moonjely et al. 2023). The release of spores in the spring leads to infection of seedling roots and stem bases driving FCR disease (Karlsson et al. 2021), while spores released at the flowering stage can infect spikes initiating FHB development (Teli et al. 2020). Therefore, to account for the role of pathogen survival in disease progression, it appears that targeting overwintering fungal inoculum is critical to achieving successful disease control.

Biological management strategies to control plant diseases appear to provide benefits through the ability of protective agents to colonize and exploit different environmental backgrounds. Mycoparasitic fungi are of particular interest due to their ability to invade and reduce pathogen inoculum (Luongo et al. 2005; Köhl et al. 2019; Sun et al. 2020; Modrzewska et al. 2022).

Selection of strains for biocontrol purposes requires rigorous taxonomical analysis. To achieve this, we determined the taxonomic status of the *Clonostachys*-like strains using multiple nuclear loci. However, we found that among nine tested barcodes, only five allowed for the determination of *C. rosea* using BLAST searches. We also found that BLAST analyses allow reliable identification of *C. solani* through only two barcodes.

Failure in resolving taxonomic issues using the remaining loci resulted from a small fraction of sequences from *Clonostachys* spp. available in the GenBank database and hypothetically due to incorrect labeling of sequence entries in GenBank. We believe that our novel genomic data deposited in GenBank database will provide a valuable source of information for further taxonomic studies of *Clonostachys* fungi using sequence-based comparisons.

In the present study, we applied various *Clonostachys* strains to substrates colonized by various *Fusarium* strains to evaluate their mycoparasitic efficiency against major *Fusarium* spp. causing cereal diseases. Our experimental approach is comparable to the natural scenario when a beneficial strain enters a substrate that was previously occupied by the pathogen (Sarrocco et al. 2021). It is worth noting, however, that this experimental design does not fully correspond to field conditions where fungi are subjected to multiple environmental variables. However, in contrast to field trials, the in vitro approach enables a more precise estimation of the mycoparasitic efficiency of fungi, which is difficult to study in environmental samples due to the low and often irregular distribution of fungi (Penton et al. 2016; Sarrocco et al. 2021).

To identify biocontrol activity, contacting colonies of the mycoparasite and host fungus are often examined through macro- and microscopic observations (Modrzewska et al. 2022). Although previous results often support the relevance of such assays in examination of the mycoparasitism, some authors indicated challenges resulting from the low activity of fungi under in vitro conditions (Schöneberg et al. 2015; Besset-Manzoni et al. 2019). For example, widely used agar media for testing the biocontrol efficiency of *Trichoderma* strains appear to be ineffective for other mycoparasites such as *C. rosea*, which shows better performance on natural substrates (Schöneberg et al. 2015). Improvement of experimental design could be achieved by the use of natural substrates, which have been shown to increase the predictability of the bioassays and are encouraged in light of the significance of crop residues as a disease reservoir (Schöneberg et al. 2015; Elena and Köhl 2020).

In this study, we proved that prior colonization of plant substrates by *Fusarium* spp. can be effectively reduced by *C. rosea*. We demonstrated that the efficiency of *C. rosea* appears to remain at a similar level for most studied strains regardless of the target pathogen. In general, our results are in line with previous studies demonstrating promising biocontrol efficiency of *C. rosea* against different *Fusarium* pathogens such as *F. avenaceum* (Palazzini et al. 2013), *F. culmorum* (Jensen et al. 2000), *F. graminearum* (Hue et al. 2009; Palazzini et al. 2013; Khairullina et al. 2023), *Fusarium oxysporum* f. sp*. radicis-cucumerinum* (Chatterton and Punja 2010), and *Fusarium verticillioides* (Palazzini et al. 2013). However, we found that a small fraction of samples, mostly bioassays with strain L99 did not reveal a significant reduction of pathogen inoculum, raising questions on strain-dependent differences in colonization efficiency. Nevertheless, such differences were not identified through visual assessment of bioassays with L99 (Fig. 2b). Further results of qPCR analyses confirmed visual observations and showed that in 17 out of 24 treatments, L99 was detected even at higher levels than Ant1 being highly effective in decreasing *Fusarium* load. We hypothesize that these exceptional results may have been impacted by incomplete degradation of pathogen DNA in the parasited host.

Finally, our results confirm the mycoparasitic potential of *C. rosea* against the major *Fusarium* pathogens of cereals in a more natural experimental design (wheat straw being colonized by *Fusarium* fungi). Most studied strains exhibited strong mycoparasitic performance irrespective of host-fungus, type, and level of colonization of substrate by pathogens. Efficient performance of local *C. rosea* strains identifies possible targets for future biological control strategies to control Fusarium diseases in cereals.

## Supplementary Information

Below is the link to the electronic supplementary material.

Supplementary file1 (XLSX 147 KB)

Supplementary file2 (XLSX 12 KB)

Supplementary file3 (XLSX 41 KB)

Supplementary file4 (XLSX 17 KB)
